# ASSESSING TELEHEALTH USE AMONG OLDER ADULTS WITH LIMITED RESOURCES DURING THE COVID-19 PANDEMIC

**DOI:** 10.1093/geroni/igac059.1983

**Published:** 2022-12-20

**Authors:** Marie Dezine, Naushira Pandya, Sashah Damier, Sweta Tewary

**Affiliations:** Nova Southeastern University - - Ft Lauderdale, FL, North Miami, Florida, United States; Nova Southeastern University - - Ft Lauderdale, FL, Fort Lauderdale, Florida, United States; Nova Southeastern University - - Ft Lauderdale, FL, Fort Lauderdale, Florida, United States; Nova Southeastern University - - Ft Lauderdale, FL, Fort Lauderdale, Florida, United States

## Abstract

The COVID-19 pandemic created new barriers to accessing primary care services, particularly among older adults who has faced barriers related to access to care, transportation, health literacy, and social isolation. Nova Southeastern University South Florida Geriatric Workforce Enhancement Program (NSU SFGWEP) partnered with primary care clinics and a local community partner to conduct wellness calls to older adult patients identified through clinic EHR. This project aimed to provide educational and telehealth support to vulnerable adults with limited resources in the Tri-County region of Florida. Wellness calls were made to determine educational and technical support needs of the older adults designated as underprivileged. We identified 44 participants to receive telehealth devices. Samsung tablets were mailed with educational resources, developed by NSU SFGWEP related to COVID-19 pandemic. The information included health, vaccine education, and instructions to access telehealth services. They had the tablets for six months. We conducted bi-weekly calls to offer peer training to access the educational materials. The participants were asked a series of questions to assess the effectiveness of the peer training support. Among participants, 36% (n=16) found the education materials impactful. Most participants, 89% (n=39), used the tablet, and 23% (n=10) reported using it daily. 11% (n=5) used it for telehealth, 7% (n=3) to connect with friends and family, and 7% (n=3) to connect with faith. This pilot project suggests that tablets were beneficial in assisting the participants in accessing education materials and resources encouraging the use of telehealth appointments and eliminating some of the social isolation.

